# Interrelationships Among Personality Traits, Depressive Symptoms, Childhood Abuse, and Social Disability

**DOI:** 10.1155/da/2250192

**Published:** 2025-10-01

**Authors:** Zizhao Feng, Jia Zhou, Rui Liu, Le Xiao, Yuan Feng, Ruinan Li, Xiaoya Li, Xueshan Zhang, Jing Liu, Gang Wang, Jingjing Zhou

**Affiliations:** ^1^The National Clinical Research Center for Mental Disorders and Beijing Key Laboratory of Mental Disorders, Beijing Anding Hospital, Capital Medical University, Beijing, China; ^2^Advanced Innovation Center for Human Brain Protection, Capital Medical University, Beijing, China

**Keywords:** loss of pleasure sensation, major depressive disorder, personality trait, psychoticism

## Abstract

**Background:**

Personality traits and childhood abuse were found to be associated with depressive symptoms and with each other. However, no previous study has elucidated the directional interrelationship among those factors in a clinical population of patients with major depressive disorder (MDD). This study sought to construct networks to explicate the directional interrelationship among those factors and social disability, identify the most central factor, and explore potentially existing causality chains implied by the directed association chains.

**Methods:**

This cross-sectional study analyzed data from 1445 patients with MDD in a national cohort. Personality traits were measured using the Eysenck Personality Questionnaire-Revised Short Scale for Chinese (EPQ-RSC). Seven dimensions of depressive symptoms were measured with various scales: depression, anxiety, insomnia, and somatic symptoms with the 17-item Hamilton Depression Rating Scale (HAMD-17), the loss of pleasure sensation with the Snaith-Hamilton Pleasure Scale (SHAPS), apathy with the Modified Apathy Evaluation Scale (MAES), and fatigue with the Chalder Fatigue Scale (CFS-11). Childhood abuse experience was measured using the Childhood Trauma Questionnaire-Short Form (CTQ-SF). Social disability was measured with the Sheehan Disability Scale (SDS). Undirected and Bayesian network analyses were used to identify central factors and explore directional interrelationships among the variables.

**Results:**

The loss of pleasure sensation was the most central in terms of strength and closeness. In the directed acyclic graph (DAG) derived from the Bayesian network analysis, psychoticism was positioned at the highest level in the model, suggesting its causal precedence. One key directed association chain, which implied a potentially existing causality chain, was that psychoticism predicted the loss of pleasure sensation, and this symptom predicted social disability.

**Conclusion:**

Loss of pleasure sensation and psychoticism might be important for future research in MDD. The appearance of psychoticism at the beginning of the directed association chain (which implied a potentially existing causality chain) involving the central factor and the characteristics of high psychoticism implied that the social/interpersonal component of the loss of pleasure sensation may be a meaningful focus of future research and intervention of MDD.

**Trial Registration:**

Chinese Clinical Trial Registry: ChiCTR2200059053

## 1. Introduction

Major depressive disorder (MDD) is a highly prevalent psychiatric disorder that imposes a heavy disease burden [1, 2]. This disorder is heterogeneous in terms of etiology [3], and various factors contribute to the development of this disorder [4, 5], making it difficult to find focal points for intervention and prevention. Psychosocial factors, along with biological factors, were found to have played an important role in MDD [4].

Researchers and theorists have tried different ways to understand and study personality. Trait theorists hold that personality can be defined by relatively stable patterns of how an individual thinks, feels, or behaves that are unique to that individual (in other words, traits) [6]. British psychologist Hans Eysenck proposed a three-factor model that describes personality with three essential dimensions (traits), namely psychoticism, neuroticism, and extroversion [7, 8]. The personality trait psychoticism was characterized by egocentricity, hostility, suspiciousness, and impersonality; neuroticism, by emotional instability and reactivity to stimuli; and extraversion, by sociability and outgoingness [7, 9]. Based on this model, Eysenck and Eysenck [10] developed the Eysenck Personality Questionnaire (EPQ) as a tool for personality assessment.

Personality influences how an individual adapts, reacts, or responds to each situation or event [6] and, therefore, might make the individual more or less susceptible to depression. Indeed, studies have found that there were associations between personality traits and the development of depressive symptoms [11–14]. On the other hand, personality is shaped by a combination of innate predispositions and external influences [15]. Childhood abuse, as a type of external influence in an individual's development, has been found to be associated with personality traits [16, 17]. In addition, adverse childhood experiences are significantly associated with depression later in life [18], and childhood abuse, as a type of adverse childhood experience, has been found to be associated with the development of depressive symptoms [17, 19]. Moreover, one study on the general population has found that each of the three had associations with the other two and that a personality trait may be a mediator between childhood abuse and depressive symptoms [20]. Another study has found that childhood abuse was both directly associated with depressive symptoms and indirectly associated with depressive symptoms through the mediation of personality [21].

The complex findings regarding relationships among those factors (personality, childhood abuse experience, and depressive symptoms) may be explained within the framework of the bioecological systems theory proposed by Bronfenbrenner [22]. This theory proposes that human development is driven by the interaction of four elements: process, person, contexts, and time. All elements interact with and might influence each other, leading to optimal or dysfunctional developmental outcomes. Indeed, there have been studies employing the bioecological systems theory as the theoretical foundation, with various factors or elements as study subjects, in the exploration of mental health outcomes including depressive symptoms [23, 24], internalizing symptoms [25, 26], and psychological distress [27, 28]. Therefore, in the current study, we also understood and connected the variables through the lens of the bioecological systems theory. Here, personality, encompassing both biological (innate predispositions) and psychosocial (the way one adapts, reacts, interprets, etc.) characteristics, is a factor of the “person” element, childhood abuse experience is a factor of the microsystem (which includes settings the individual directly interacts with, such as family, friend group, or workplace [22]) of the “systems” element, and dimensions of depressive symptoms can be viewed as dysfunctional developmental outcomes. As the theory suggests that the factors might influence each other and directly or indirectly lead to the outcomes, it may be meaningful to illustrate the interrelationship among the factors and elucidate the relative importance of each factor in the network.

The previous studies suggested that both personality traits and childhood abuse may be factors directly or indirectly associated with depressive symptoms. Moreover, the bioecological systems theory suggests that there may be complex interrelationships among those variables that call for an exploration of all possible associations. However, to the best of our knowledge, no previous study has elucidated the directional interrelationship among specific personality traits, childhood abuse, and specific depressive symptoms in a clinical population of patients with MDD and further explored potentially existing causality chains among them. Therefore, the current study sought to construct undirected and Bayesian networks that include those factors (three personality traits, seven dimensions of depressive symptoms, and childhood abuse) and social disability (given that social disability is a pervasive impairment in patients with MDD [29]) in order to explicate the interrelationship among them and to identify the most central factor (for instance, either paricular personality trait, particular symptom, or childhood abuse experience) which may be the target for prevention and intervention of MDD. Further, this study sought to explore the directional associations (which imply potentially existing causal relationships) among the factors with the Bayesian network, as each ring of the causality chain implies potential focal points of future studies and intervention.

## 2. Methods

### 2.1. Participants

This study used data from the Prospective Cohort Study of Depression in China (PROUD), an ongoing, nationally representative, multicenter cohort study planned to be conducted from January 2022 to December 2026 in 52 qualified tertiary hospitals in China. All procedures in the study complied with the ethical standards of the Declaration of Helsinki. Ethical approvals were obtained from The Medical Ethical Committee of Beijing Anding Hospital, Capital Medical University (Approval Number 14. [2022] keyan-202221FS-2), as well as all independent ethics committees. Written informed consents were obtained from all participants. The baseline data of 1445 eligible patients with MDD analyzed in this study were collected from May 12, 2022, to June 30, 2024, in 18 qualified tertiary hospitals located in 13 provinces and cities of China (i.e., municipalities Beijing, Tianjin, and Shanghai, as well as provinces Sichuan, Zhejiang, Anhui, Hebei, Henan, Yunnan, Guangdong, Shandong, Shaanxi, and Hunan).

To be included in the PROUD study, an individual needed to: (1) be diagnosed with MDD by a clinician using the Mini-International Neuropsychiatric Interview (MINI), (2) be 18–65 years old, (3) have a score of 14 or more on the 17-item Hamilton Depression Rating Scale (HAMD-17) both at screening and baseline, (4) have no antidepressants taken in at least 14 days before screening, and (5) be planned to be treated with antidepressant monotherapy. Individuals would be excluded if they had psychiatric comorbidities or other medical conditions that would obstruct them from completing the study. More details can be found in the published protocol for the PROUD study [30].

### 2.2. Measures

Personality traits were measured using the EPQ-Revised Short Scale for Chinese (EPQ-RSC), a translated and revised version of the EPQ-Revised Short Scale (EPQ-RS). The EPQ-RSC is a self-report scale with 48 questions that are supposed to be answered with “yes” or “no.” It consists of four dimensions with 12 questions in each (psychoticism measured with questions 2, 6, 10, 14, 18, 22, 26, 28, 31, 35, 39, and 43, neuroticism measured with questions 1, 5, 9, 13, 17, 21, 25, 30, 34, 38, 42, and 46, extraversion measured with questions 3, 7, 11, 15, 19, 23, 27, 32, 36, 41, 44, and 48, and a lie detector inventory); the score of each dimension is calculated by summing the 12 item scores. This scale has been validated in Chinese adults [31]. Personality variables in the current study were the level of psychoticism, neuroticism, and extraversion reflected by corresponding dimension scores.

Seven dimensions of depressive symptoms were included in the current study: depression, anxiety, insomnia, and somatic symptoms were measured with the Chinese version [32] of the HAMD-17 [33], a clinician-rated scale with 17 items scored from 0 to 2 or from 0 to 4 (higher scores indicate greater symptom severity). The scale showed satisfactory psychometric properties in Chinese patients with depression (Cronbach's *α* = 0.714) [32]. Following previous methodology [34], scores of certain items were summed to measure depression (items 1, 2, 3, 7, and 8), anxiety (items 9, 10, 11, 15, and 17), insomnia (items 4, 5, and 6), and somatic symptoms (items 12, 13, 14, and 16) in the current study.

The fifth dimension of depressive symptoms, the loss of pleasure sensation, was measured with the Chinese version [35] of the Snaith-Hamilton Pleasure Scale (SHAPS) [36], a 14-item self-report checklist. Items of this scale cover four domains of hedonic experience; each item is scored from 1 (definitely agree) to 4 (definitely disagree), yielding a total score ranging from 14 to 56. A higher score indicates a higher level of loss of pleasure sensation. The scale has been validated in Chinese clinical samples (Cronbach's *α* = 0.93) [35]. The current study focused on the level of overall loss of pleasure sensation reflected by the total score of the scale.

The sixth dimension of depressive symptoms, apathy, was measured with the Chinese version of the Modified Apathy Evaluation Scale (MAES) [37], a 14-item scale measuring the level of apathy. Each item is scored from 0 to 3; for items 1–8, 0 represents “a lot” and 3 represents “not at all,” but for items 9–14, 0 represents “not at all” and 3 represents “a lot.” The total score ranges from 0 to 42, with a higher score indicating a higher level of apathy. The current study focused on the level of overall apathy reflected by this total score.

The seventh dimension of depressive symptoms, fatigue, was measured with the Chinese version [38] of the Chalder Fatigue Scale (CFS-11) [39], an 11-item self-report scale measuring fatigue in the physical and mental domains. Each item is scored from 0 to 3, and a total score is calculated by summing all item scores, with a higher score indicating greater fatigue. This scale has been validated in a Chinese community sample, with a Cronbach's *α* of 0.86 [38]. The current study focused on the level of overall fatigue reflected by the total score.

Childhood abuse experience was measured using the Chinese version [40] of the Childhood Trauma Questionnaire-Short Form (CTQ-SF) [41], a brief, self-report questionnaire covering emotional, physical, and sexual abuse, as well as emotional and physical neglect. The scale consists of 28 items rated on a 5-point Likert scale with item scores from 1 (“never”) to 5 (“always"). Items 2, 5, 7, 13, 19, 26, and 28 are reverse-scored. The total score ranges from 25 to 125, with higher scores indicating a higher level of childhood abuse experience. The scale has been validated in Chinese patients with depression (Cronbach's *α* = 0.81) [40]. The current study focused on the overall childhood abuse experience reflected by the total score.

Social disability was measured with the Chinese version [42] of the Sheehan Disability Scale (SDS) [43], a self-report scale measuring disabilities in work/school, social life, and family life domains. Each domain is rated on a visual analog scale ranging from 0 (“no impairment”) to 10 (“extreme”). The total score ranges from 0 to 30, with a higher score indicating greater social disability. This scale has demonstrated good psychometric properties in the Chinese population (Cronbach's *α* = 0.94) [42]. The current study focused on the overall social disability reflected by the total score.

### 2.3. Statistical Analysis

We used the Monte Carlo simulation method that was proposed recently to conduct the sample size estimation for this network analysis [44]. Employing this method, we first optimized the outcome (sensitivity = 0.8) and statistical power (specificity = 0.8). Then, we performed the Monte Carlo simulations (*n* = 30) to calculate these parameters across putative sample sizes, with curve-fitting methods to estimate these statistics. Finally, to determine the sample size, the uncertainty bounds were estimated around these curves using the bootstrapping method. These analyses were validated as well. These sample size analyses have been carried out using the Powerly package in R. We found that 344 participants were sufficient to meet the analytical requirements for the present study, and our sample size of 1445 exceeds this necessary amount. Details are presented in Figures S1–S4 in the Supporting Information.

Descriptive statistics were presented as medians with interquartile ranges (IQRs) for continuous variables and as counts with percentages for categorical variables.

First, carried out with the R-package, the Extended Bayesian Information Criterion Graphical Lasso (EBICglasso) method [45, 46] was used to construct a partial correlation network that included: (1) three personality traits (the level of psychoticism, neuroticism, and extraversion measured with corresponding dimension scores of the EPQ-RSC); (2) seven dimensions of depressive symptoms (the level of depression, anxiety, insomnia, and somatic symptoms measured with the sum of corresponding item scores on the HAMD-17, the level of the loss of pleasure measured with the SHAPS total score, the level of apathy measured with the MAES total score, and the level of fatigue measured with the CFS-11 total score); (3) childhood abuse (measured with the CTQ-SF total score); and (4) social disability (measured with the SDS total score). In this network, each node represents a variable, and the thickness of each edge reflects the magnitude of the regularized partial correlation between a variable pair. Thicker, more saturated edges indicate stronger correlations, and thinner edges indicate weaker correlations. Green edges represented positive associations, and red edges represented negative associations.

Next, centrality measures (namely strength, betweenness, and closeness) of all nodes were calculated to assess the relative importance and influence of the nodes in the network [47]. Strength of a node, a common and stable centrality metric, was computed by summing the weights of all edges connected to the node. Betweenness of a node was determined by counting the number of the shortest paths passing through that node. Closeness of a node was defined as the inverse of the sum of distances from a node to all other nodes in the network. Variables with higher centrality scores, particularly those with higher strength, have a greater influence on the other variables in the network [48]. The presence of these highly central variables increases the likelihood of the appearance of other connected variables.

Third, to assess the stability and accuracy of the network, bootstrapping procedures were carried out using the R package bootnet. This process included: (a) estimating bootstrapped confidence intervals (CIs) for edge weights by calculating their 95% CIs through a nonparametric bootstrap approach (*n* = 1000); (b) evaluating the stability of centrality indices with a case-drop bootstrap method, where centrality values were repeatedly calculated from subsets of the data with increasing proportions of cases removed. The correlation stability (CS) coefficient, a measure of the maximum proportion of cases that could be removed while maintaining a correlation of 0.7 in at least 95% of the sample, was used to quantify the stability of these centrality indices. A CS coefficient above 0.5 was considered acceptable, and CS coefficients below 0.25 indicated poor stability; (c) conducting bootstrapped difference tests (*α* = 0.05) for edge weights and node strengths using 1000 bootstrap samples. Gray boxes denoted nodes that did not significantly differ from one another, while black boxes indicated nodes with significant differences.

Fourth, a Bayesian network, grounded in causal reasoning principles, was constructed by combining a directed acyclic graph (DAG) with a probability distribution [49]. The DAG captures conditional independence relationships and encodes the joint probability distribution of the variables using graphical separation. As outlined in Pearl's work [50], DAGs are useful for representing hypothetical causal structures. Although causality cannot be conclusively determined from observational data alone, DAGs offer invaluable insights into the direction and strength of relationships among variables.

This approach has become increasingly prevalent in psychological research [51]. For the Bayesian network estimation, the R package bnlearn was used. The algorithm employed a hill-climbing approach [52], as described in recent studies [53]. To ensure the stability of the final network, a bootstrapping procedure with 1000 iterations was performed. The Bayesian Information Criterion (BIC) was calculated for each sample as a goodness-of-fit index. Edges that maintained the same direction in at least 85% of the bootstrapped networks were retained in the final model [54]. All statistical analyses were conducted using R version 4.4.1 (R Foundation for Statistical Computing, Vienna, Austria).

## 3. Results

A total of 1445 participants were included in the study, of which 34.53% were male. The median age was 27.84 years (IQR: 22.95–35.87). The majority of participants (94.02%) were of Han ethnicity. The mean duration of illness was 52.00 months (range: 28.00–94.00), and 59.17% (*n* = 855) of the participants were experiencing their first episodes. Demographic and clinical characteristics of the study population are summarized in Table 1. The mean scores of the HAMD-17 dimensions, CTQ-SF, SDS, EPQ-RSC, SHAPS, MAES, and CFS-11 are reported in Supporting Information Table S1.

**Figure 1:**
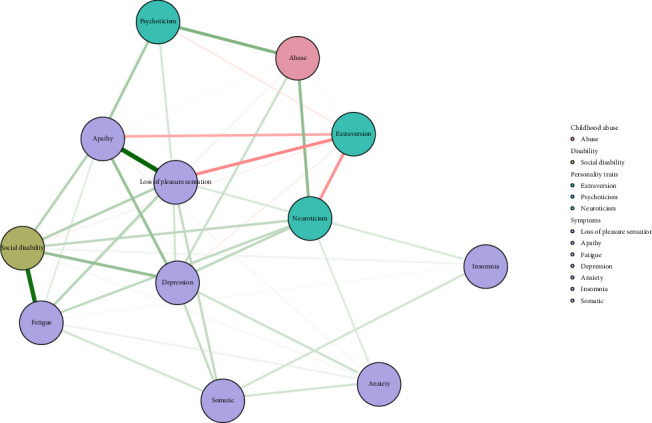
Undirected network of personality traits, depressive symptoms, childhood abuse, and social disability in patients with MDD. Note: Each edge corresponds to a correlation (positive in green and negative in red) between two nodes. The thickness of the edge corresponds to the absolute magnitude of the correlation.

The network structure consisted of 12 nodes (Figure 1). Most edges between symptoms and extraversion indicated negative correlations, whereas all edges involving neuroticism were positive. The node abuse showed strong connections with both psychoticism and neuroticism. The most prominent edges were observed between the nodes the loss of pleasure sensation and apathy, as well as between fatigue and social disability. Among the depressive symptoms, strong associations were found between depression and both apathy and social disability.

**Figure 2:**
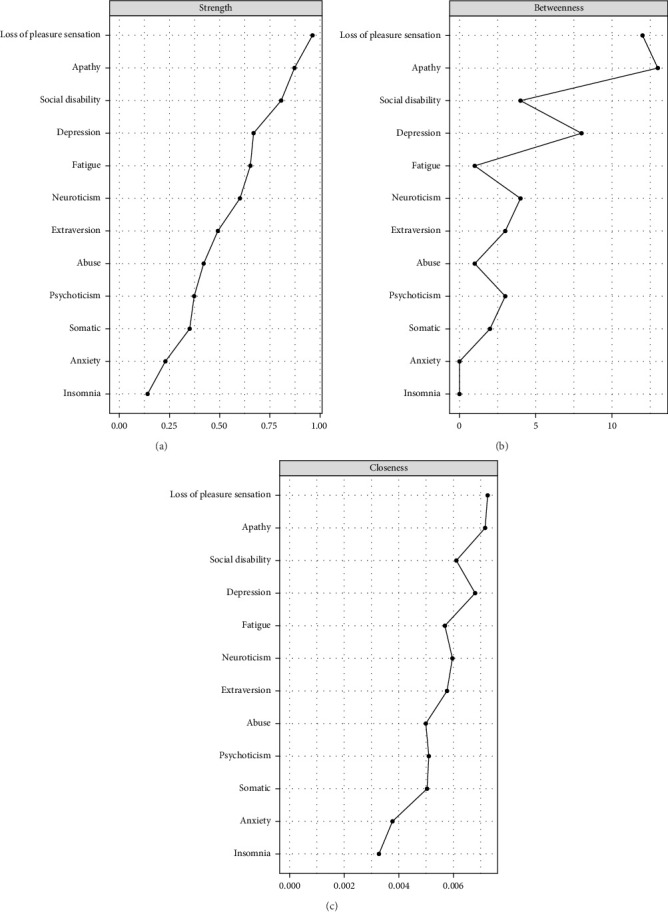
Node centrality metrics of the network. The (a), (b), and (c) show the strength, betweenness, and closeness estimates for each node of the network, respectively.

Three centrality metrics—strength, betweenness, and closeness—are shown in Figure 2. A node with a higher centrality index is more central within the network, implying stronger connections to other variables. The loss of pleasure sensation exhibited the highest strength centrality, indicating that it had the strongest direct connections to other variables. Apathy emerged as the most central node in terms of betweenness, suggesting that it frequently served as a bridge for information flow across the network. The loss of pleasure sensation also had the highest closeness centrality, indicating that it was the key node most likely to rapidly influence other variables.

**Figure 3:**
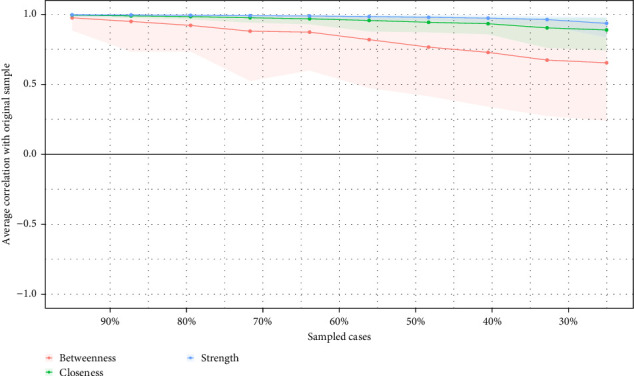
Stability of the centrality indices: point estimates and corresponding 95% CIs. Note: This was determined by average correlations between the centrality indices of networks sampled with patients dropped and the original sample. Lines indicate the means, and areas indicate the range from the 2.5th quantile to the 97.5th quantile.

Data from the case-drop procedure indicated a reliable estimation of centrality (Figure 3). The correlations between the centrality measures calculated from the full sample and a 30% subset exceeded 0.25, suggesting that the network structure remained stable even with 70% of the sample randomly removed. The bootstrapped 95% CIs for edge weights are presented in the Supporting Information (Figure S5). Additionally, the network demonstrated stability across bootstrapped difference tests for both edge weights and node strength (online Supporting Information Figures S6 and S7).

**Figure 4:**
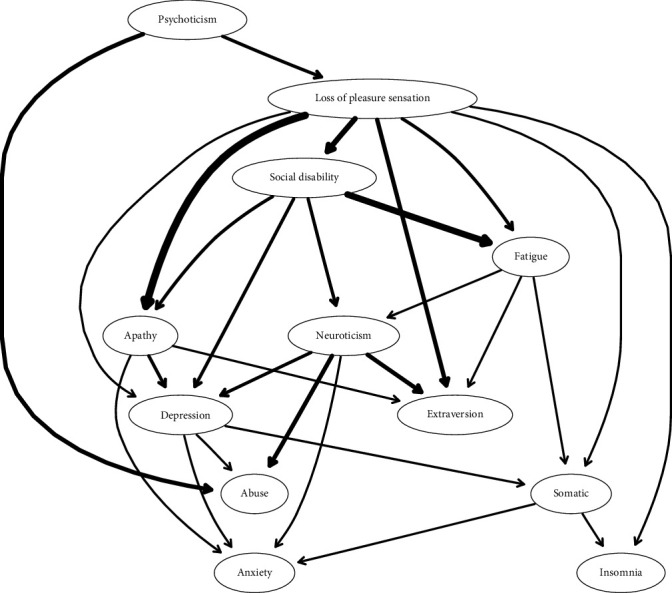
Directed acyclic graph (DAG) illustrating relationships among personality traits, depressive symptoms, childhood abuse, and social disability in patients with MDD. Note: Arrows indicate the direction of the assumed causal relationships. Edge thickness indicates the confidence in the direction of prediction shown.

Figure 4 shows the DAG derived from the Bayesian network analysis, illustrating the relationships among childhood abuse, personality traits (i.e., extraversion, psychoticism, and neuroticism), depressive symptoms (i.e., loss of pleasure, apathy, fatigue, depression, anxiety, insomnia, and somatic symptoms), and social disability in patients with MDD. The thicknesses of the edges reflect the strengths of the connections in the network, while the position of each node indicates its predictive priority. Psychoticism is positioned at the highest level in the model, suggesting its causal precedence. It predicts the activation of the loss of pleasure sensation which in turn triggers social disability, followed by fatigue, somatic symptoms, and insomnia. Another key pathway involves psychoticism to the loss of pleasure sensation which activates apathy, depression, and anxiety. As shown in the regularized partial correlation network, some nodes, such as apathy, neuroticism, and depressive symptoms, act as “bridges” between these pathways.

## 4. Discussion

The current study illustrated the interrelationship among personality traits, childhood abuse, depressive symptoms, and social disability in a clinical population of patients with MDD with undirected and Bayesian network analyses. It identified the most central factors in the entire network and further elucidated the directional associations among the factors with the DAG.

The undirected network analysis revealed that the symptom loss of pleasure sensation was central in the entire network and had connections to the largest number of factors. Moreover, the DAG revealed that it predicted all other depressive symptoms directly or indirectly, and it was also the only symptom that directly predicted social disability. Among all directional associations depicted by the DAG that implied potentially existing causal relationships that could be tested in future studies, a key potential causality chain we discovered was that the personality trait psychoticism predicted the symptom loss of pleasure sensation, and this symptom, in turn, predicted, directly or indirectly, social disability and all other depressive symptoms. These chains echoed the undirected network constructed with the EBICglasso method [45, 46] in which psychoticism was positively correlated with the loss of pleasure sensation and the loss of pleasure sensation was correlated with almost all other depressive symptoms. On the other hand, unexpectedly, the DAG revealed that personality traits neuroticism and psychoticism and the symptom depression predicted childhood abuse, instead of vice versa, which we interpreted as reflecting associations of personality and depression with the reporting of childhood abuse, instead of with the abuse experience itself.

Loss of pleasure sensation, or anhedonia, refers to a diminished pleasure or interest in all or most usual activities [55]. It has been viewed as one of the core symptoms of MDD: the Diagnostic and Statistical Manual of Mental Disorders, the fifth edition (DSM-5), prescribed that at least one of the symptoms, “depressed mood” or “loss of interest or pleasure,” must exist for an individual to be diagnosed with MDD [55]. Our finding of the level of the loss of pleasure sensation as central in the network was consistent with this common view.

A meaningful finding was the appearance of psychoticism at the beginning of the directed association chain (which implies a potentially existing causality chain) involving the loss of pleasure sensation, the central symptom of MDD, which implied the important role of psychoticism in MDD. First of all, because psychoticism is characterized by eccentric behavior and quasi-psychotic experiences, it is conventionally associated with schizophrenia-spectrum disorders instead of mood disorders [56]. Although previous studies have found that both psychoticism and neuroticism were related to depressive traits or symptoms [17, 56], mainstream studies of mood disorders still took neuroticism as the main focus [57], as neuroticism, by definition, is related to emotional reactivity [7]. However, our findings suggested that the role of psychoticism in MDD may be overlooked, and psychoticism might be worth attention in future studies of MDD. Second, as scholars have mentioned, personality traits may be intermediate phenotypes that provide more tractable targets than clinical diagnoses for research on underlying genetic and neurobiological mechanisms [3, 58]. Eysenck, in proposing his three-factor model, also emphasized and validated that there were biological mechanisms (for instance, brain activities, neural activities, and hormonal balances) underlying his proposed personality traits that could distinguish the traits from each other [7, 9]. Therefore, our finding suggest that turning attention to the biological characteristics of individuals with elevated psychoticism may help clear the fog around the underlying mechanism of anhedonia in MDD. Third, as previously mentioned, personality traits influence the patterns of behavior and thinking of an individual. Therefore, future studies could also explore possible psychological mechanisms of anhedonia through the patterns (for instance, coping styles or strategies) associated with high psychoticism, which may also help to identify the targets for psychotherapeutic interventions.

Currently, there is still no consensus regarding the underlying mechanism of the central symptom anhedonia, making it difficult to determine a target for intervention. Researchers have offered many different hypotheses, but even the most acknowledged hypothesis of the role of dopamine lacks conclusive empirical support [59]. The inconsistency in findings regarding the mechanism may be a result of the inconsistent and underspecified definition of anhedonia that might have led researchers to study different components of it [59]. Our finding of the directed association chain (which implies a potentially existing causality chain) with psychoticism predicting anhedonia and anhedonia predicting social disability suggested a potential focus for future studies on anhedonia in MDD. High psychoticism is often related to maladjustment in social or interpersonal aspects, characterized by hostility, impersonality, and lack of empathy [9]. Correspondingly, a previous study revealed that a lack of empathy was related to anhedonia [60]. Another study using network analysis to investigate the relationship between risk features of psychiatric disorders and anticipatory pleasure deficits discovered that interpersonal features of schizotypal personality traits had the greatest impact in the whole network, and social anticipatory pleasure had the greatest impact among all anticipatory pleasure components [61]. In another study, individuals with a high level of social anhedonia showed generalized pleasure deficits [62]. Furthermore, it was revealed that individuals with social anhedonia may have reduced motivation and effort level regarding social activities (echoing our findings of the loss of pleasure sensation predicting apathy and fatigue, as shown in the DAG), which might explain the directional association we found between anhedonia and social disability [63]. Therefore, the overlap in the social/interpersonal component might explain our findings of psychoticism at the beginning of the directed association chain. Those findings, taken together, provided circumstantial evidence for the weight of the social and interpersonal component of anhedonia in the overall anhedonia symptom in MDD, suggesting a possible focus of future research and intervention that may be overlooked currently.

Other notable points are the counterintuitive and unexpected findings that personality traits neuroticism and psychoticism and the symptom depression seemingly predicted childhood abuse instead of vice versa. The directions of the associations are temporally illogical: childhood abuse experiences happened in the past, but the level of symptoms and personality traits are measured in adulthood and only reflect the participants' state at the time of the measurement (even though personality is comparatively stable, it is not completely fixed and still changes over lifetime [6], meaning that one cannot assume that the current assessment of personality traits fully represents the condition at an earlier time). It is illogical that a later condition predicts an earlier one. It is possible that our analysis got these results because what we could measure was actually the reports of childhood abuse, but not necessarily childhood abuse experience itself. The more plausible interpretation is that these directional associations imply the possible influences of personality traits and depression on the reporting of childhood abuse. This interpretation is consistent with previous findings. Researchers have pointed out that self-reports of childhood abuse might be subject to response biases, and the measurement tool CTQ had a possible vulnerability to distortion [64]. It was found that high neuroticism increased the retrieval of memories of negative personal events [65]. Also, high psychoticism was characterized by mistrust and quasi-psychotic experiences (for instance, unusual beliefs) [56], which might influence the interpretation and report of past events. Moreover, studies have found that depression was associated with negative memory bias, including a tendency to recall negative memories more easily [66]. The significance of this finding lies in the fact that it might have exposed a common issue that calls for caution in both research and clinical practice involving childhood abuse experience. It implies the importance of integrating multiple information resources, such as medical records, police records, or reports from people around the individual evaluated, along with self-report instruments to acquire accurate information on childhood abuse, especially that of individuals with high neuroticism or psychoticism or individuals scoring high on the symptom depression.

One strength of the study is the multicenter, representative, and relatively large cross-sectional sample. The other strength is that, instead of using hypothesis modeling that tests a limited number of hypotheses made by researchers, this study used causal modeling which more comprehensively explored all possible relationships among the factors and further explored all directional associations among them. Those directional associations implied potentially existing causal relationships, offering important clues for making and testing hypotheses in future studies [67]. However, it also has limitations as many measurements relied on self-reports. Previous studies have revealed that the accuracy of self-reports on childhood abuse and an individual's self-evaluation may be impacted by various factors [64, 68]. Future studies may benefit from an integration of multiple types of measurement tools and information resources. Another limitation was that this study used cross-sectional data. It should be noted that although the directional associations provide meaningful information, causality was not directly determined with the DAG using cross-sectional data. The results should be carefully interpreted, and the potentially existing relationships still need to be tested in future studies.

## 5. Conclusion

This study constructed undirected and Bayesian networks including three personality traits, seven dimensions of depressive symptoms, childhood abuse, and social disability in a clinical population of patients with MDD and explored the directional relationships among those factors. With the network analyses, this study found that the symptom loss of pleasure sensation was the most central in the entire network. Also, there was a directed association chain (which implies a potentially existing causality chain) of the personality trait psychoticism predicting the loss of pleasure sensation and this symptom directly or indirectly predicting social disability and all other depressive symptoms. The findings of this study revealed that the symptom loss of pleasure sensation and the trait psychoticism might be important for the research in MDD and implied that the social/interpersonal component of anhedonia may be a meaningful focus of future research and intervention of MDD.

## Figures and Tables

**Table 1:** Basic information.

Variables	*n* (%) or median (IQR)
Age	27.84 (22.95–35.87)
Sex	
Male	499 (34.53)
Female	946 (65.47)
Ethnicity	
Han	1351 (94.02)
Hui	26 (1.81)
Manchu	12 (0.84)
Mongolian	11 (0.77)
Other	37 (2.57)
Educational level	
Elementary school	26 (1.81)
Middle school	127 (8.86)
High school	183 (12.76)
Undergraduate school	913 (63.67)
Postgraduate school	185 (12.90)
Location	
Urban	1183 (85.29)
Rural	204 (14.71)
Monthly income per household (CNY)	
1000 And below	35 (2.63)
1001–5000	376 (28.23)
5001–10,000	496 (37.24)
10,000 And above	425 (31.91)
Marital status	
Unmarried	876 (61.22)
Married	475 (33.19)
Divorced or widowed	80 (5.59)
Insurance	
Urban resident basic medical insurance	305 (22.53)
Urban employee basic medical insurance	625 (46.16)
Publicly funded	72 (5.32)
Self-funded	144 (10.64)
Cooperative medical care	183 (13.52)
Other	25 (1.85)
Employment status	
Waiter/waitress	17 (2.30)
Administrative personnel	25 (3.39)
Worker	62 (8.40)
Company employee	350 (47.43)
Civil servant	44 (5.96)
Teacher	44 (5.96)
Soldier/military personnel	2 (0.27)
Researcher/scientist	12 (1.63)
Farmer	7 (0.95)
Businessperson/entrepreneur	39 (5.28)
Medical worker/healthcare worker	30 (4.07)
Others	106 (14.36)
First episode	855 (59.17)
Duration of total course (month)	52.00 (28.00–94.00)
Duration of current episode (month)	2.00 (1.00–8.50)
Onset age (year)	27.00 (21.00–35.00)

## Data Availability

The data that support the findings of this study are available from the corresponding author upon reasonable request.

## Supporting Information

Supporting Information The Supporting Information contains the following table and figures: 1. Table S1. The mean scores for different variables. 2. Sample size calculation. 2.1 Figure S1. Monte Carlo simulations for performance and statistical power. 2.2 Figure S2. Curve-fitting statistics of different sample sizes. 2.3 Figure S3. Estimates of confidence of sample sizes to fulfill required statistics. 2.4 Figure S4. Results of sample size estimates. 3. Robustness analyses. 3.1. Figure S5. Edge weight stability. 3.2. Figure S6. Edge weight differences at baseline. 3.3 Figure S7. Strength differences at baseline.
