# Supplementary material for: Interrelationships Among Personality Traits, Depressive Symptoms, Childhood Abuse, and Social Disability
Source: Depress Anxiety. 2025 Oct 1;2025:2250192. doi: 10.1155/da/2250192 (PMC12507496; doi:10.1155/da/2250192)
Supplement: Supporting Information — The Supporting Information contains the following table and figures: 1. Table S1. The mean scores for different variables. 2. Sample size calculation. 2.1 Figure S1. Monte Carlo simulations for performance and statistical power. 2.2 Figure S2. Curve-fitting statistics of different sample sizes. 2.3 Figure S3. Estimates of confidence of sample sizes to fulfill required statistics. 2.4 Figure S4. Results of sample size estimates. 3. Robustness analyses. 3.1. Figure S5. Edge weight stability. 3.2. Figure S6. Edge weight differences at baseline. 3.3 Figure S7. Strength differences at baseline. [file 2250192.f1.docx]

Supplementary materials for manuscript

a The National Clinical Research Center for Mental Disorders & Beijing Key Laboratory of Mental Disorders, Beijing Anding Hospital & the Advanced Innovation Center for Human Brain Protection, Capital Medical University, Beijing, China

Contents:

1. Table1 The mean scores for different variables

2. Sample Size Calculation

2.1 Figure S1. Monte Carlo simulations for performance and statistical power

2.2 Figure S2. Curve-fitting statistics of different sample sizes

2.3 Figure S3. Estimates of confidence of sample sizes to fulfill required statistics

2.4 Figure S4. Results of sample size estimates

3. Robustness analyses

3.1. Figure S5. Edge weight stability

3.2. Figure S6. Edge weight differences at baseline

3.3 Figure S7. Strength differences at baseline

Table 1 The mean scores for different variables

| Variables | Median (IQR) |
| --- | --- |
| CTQ total score | 44.00(35.00-54.00) |
| EPQ- extraversion score | 4.00(2.00-6.00) |
| EPQ- psychoticism score | 3.00(2.00-4.00) |
| EPQ- neuroticism score | 10.00(8.00-11.00) |
| SDS total score | 16.00(10.00-21.00) |
| SHAPS total score | 33.00(30.00-37.00) |
| MAES total score | 24.00(19.00-29.00) |
| CFS-11 total score | 20.00(17.00-24.00) |
| HAMD-17-depression score | 8.00(7.00-10.00) |
| HAMD-17-anxiety score | 6.00(4.00-7.00) |
| HAMD-17-insomnia score | 4.00(2.00-5.00) |
| HAMD-17-somatic score | 3.00(2.00-5.00) |

*Note: abbreviations, Eysenck Personality Questionnaire (EPQ), Childhood Trauma Questionnaire-Short Form (CTQ-SF), 17-item Hamilton Depression Rating Scale (HAMD-17), Snaith-Hamilton Pleasure Scale (SHAPS), Modified Apathy Evaluation Scale (MAES, for apathy) and Chalder Fatigue Scale (CFS-11, for fatigue), and Sheehan Disability Scale (SDS).*

2. Sample Size Calculation


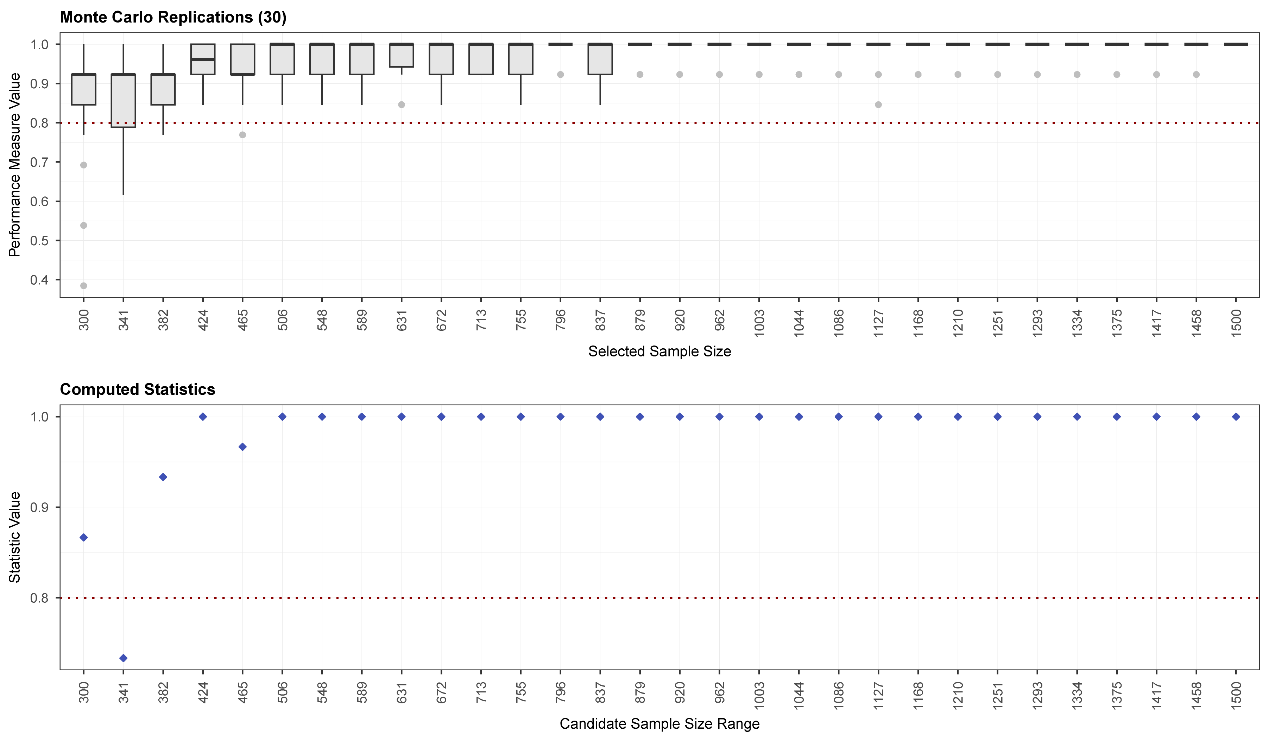


Fig S1. Monte Carlo simulations for performance (outcome, upper panel) and statistical power (lower panel).

*In the upper panel, each column shows the model performances of a given sample size across 30 Monte Carlo replications. The dashed red line indicates the acceptable criterion for determining the optimal model performance. In the lower panel, each blue point indicates the statistical value from the network model by a given sample size. The dashed red line indicates an acceptable criterion for determining the optimal model performance.*


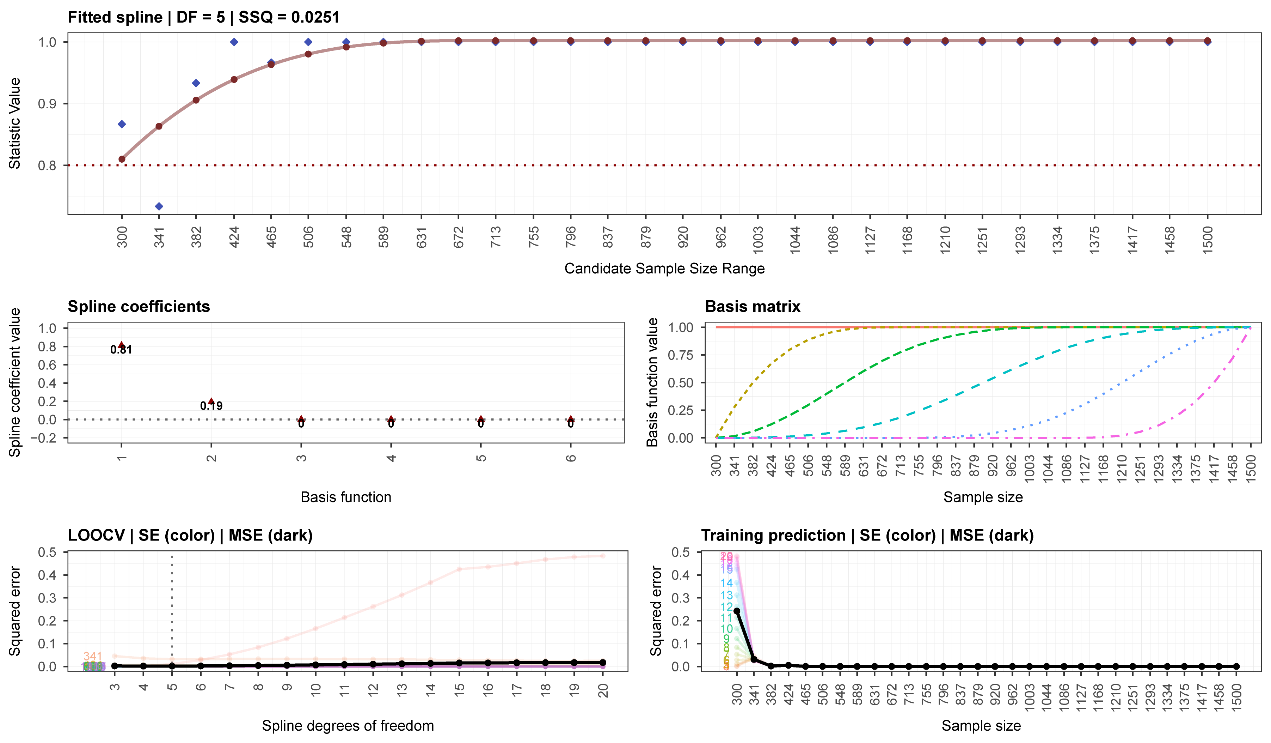


Fig S2. Curve-fitting statistics of different sample sizes.

*In the upper panel, each blue point indicates the statistical value from the network model by a given sample size. The dashed red line indicates an acceptable criterion for determining the optimal model performance. The* *translucent red line indicates the optimal curve-fitting. In the middle left panel, the spline coefficients (marked by the red triangles) are presented by varying from different basis functions. In the middle right panel, each line represents a basis function showing the changes in values across sample sizes. Different colors are used to represent different basis functions. In the lower left panel, the squared errors are estimated across different spline degrees of freedom. Different colors are used to represent different sample sizes. In the lower right panel, different colors are used to represent spline degrees of freedom of different sample sizes.*


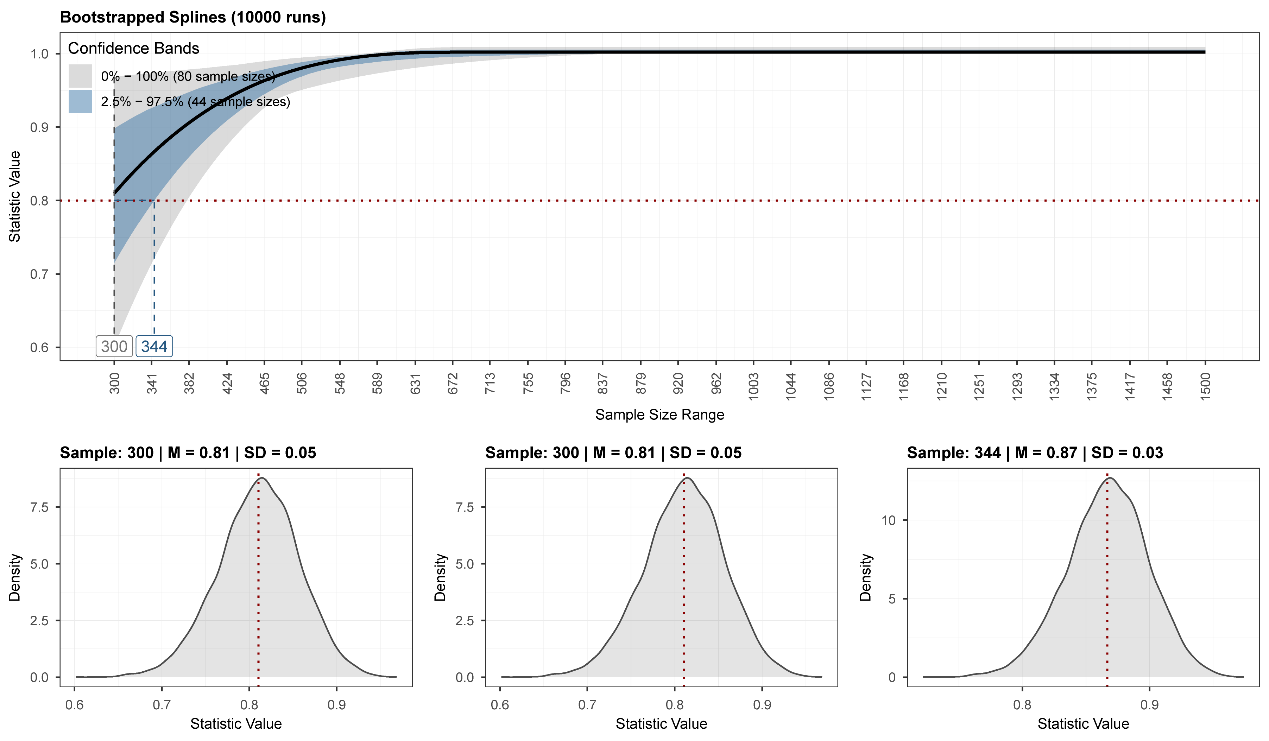


Fig S3. Estimates of confidence of sample sizes to fulfill required statistics.

*In the upper panel, the blue (gray) shadow pictured the 95% (100%) confidence bands of statistics from the network model across Bootstrapping pseudo-samples. The dashed red line indicates the acceptable criterion for determining the optimal model performance. In the lower panel, the red dashed line indicates the optimal density of statistics from the network model of given sample sizes.*


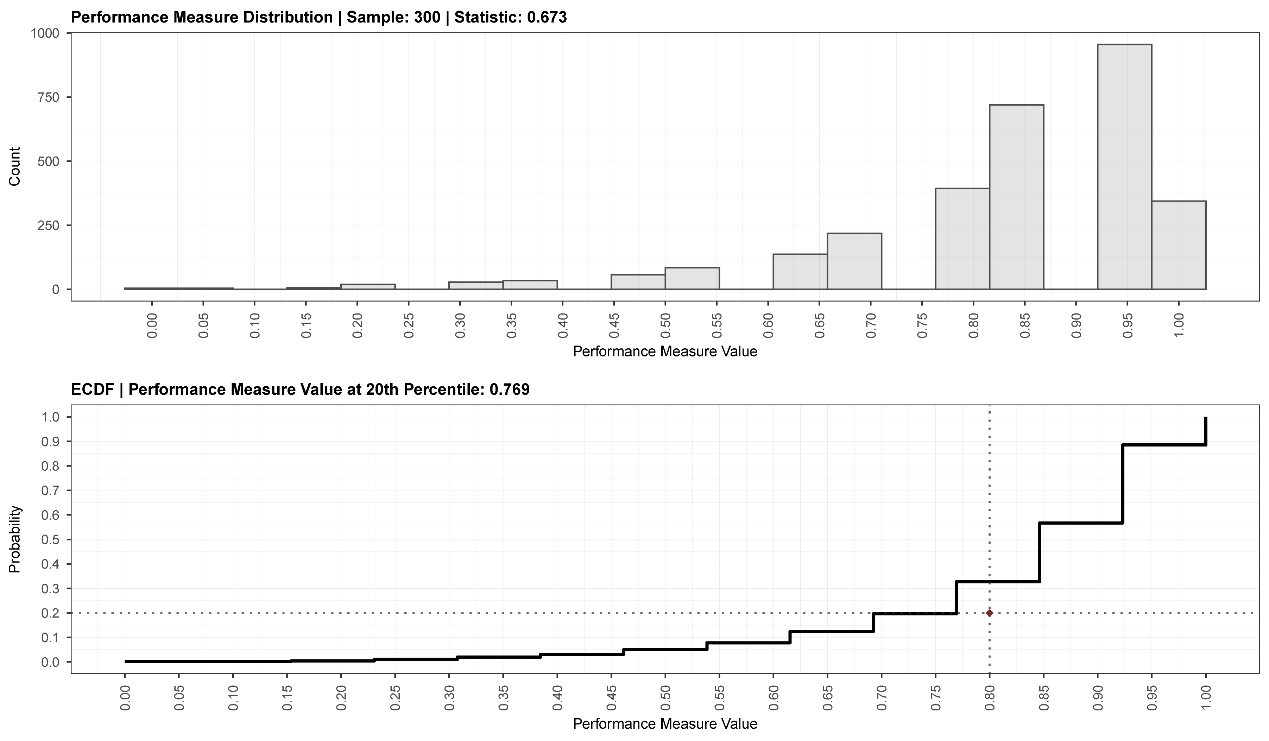


Fig S4. Results of sample size estimates.

*In the lower panel, the black solid curve indicates the probability of model performances by the Empirical Cumulative Distribution Function (ECDF). The red point depicts the cutoff of this curve.*

3. Robustness analyses

All robustness analyses were conducted using the bootnet package for R. An accessible tutorial paper for the procedures has been published (Epskamp and Fried, 2018).


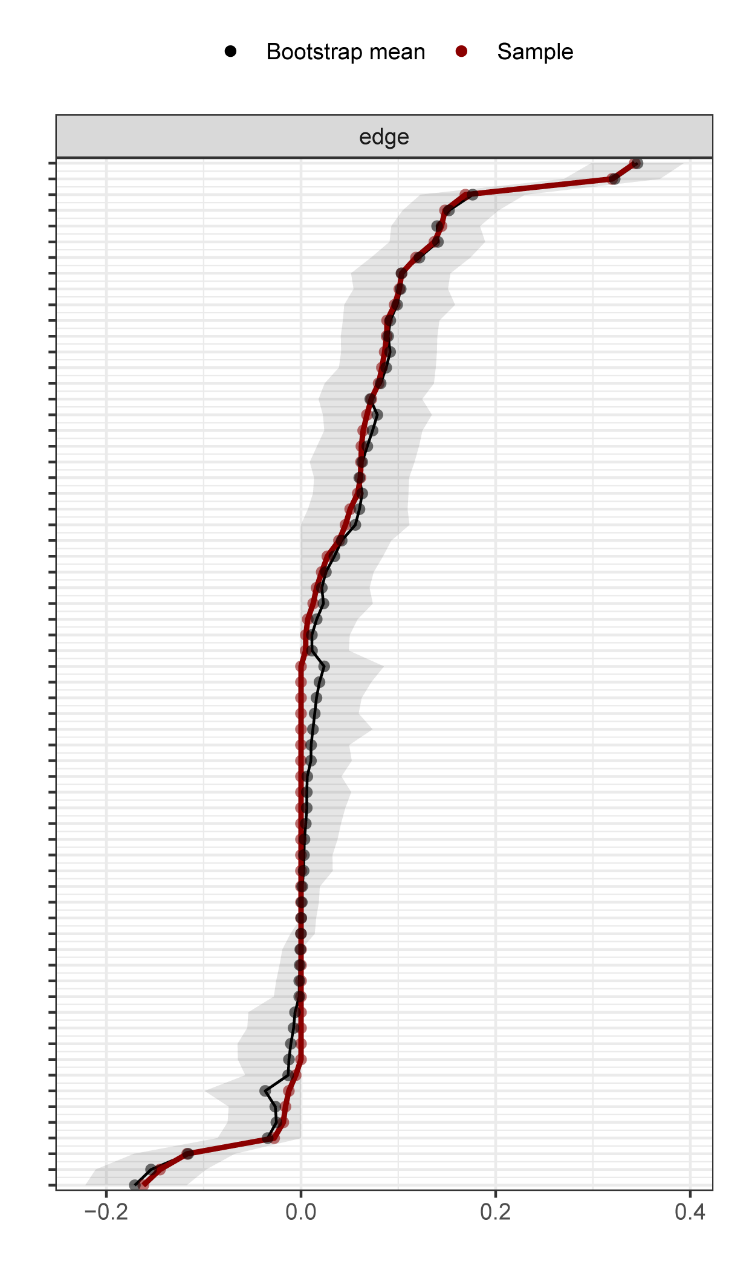


Figure S5. Edge weight stability

*Note: Red line indicates sample means, black line indicates bootstrapped means and grey area indicates 95% confidence intervals for individual edges. Narrower intervals suggest more precise estimates and overlap of intervals suggests statistically non-significant differences between edges.*


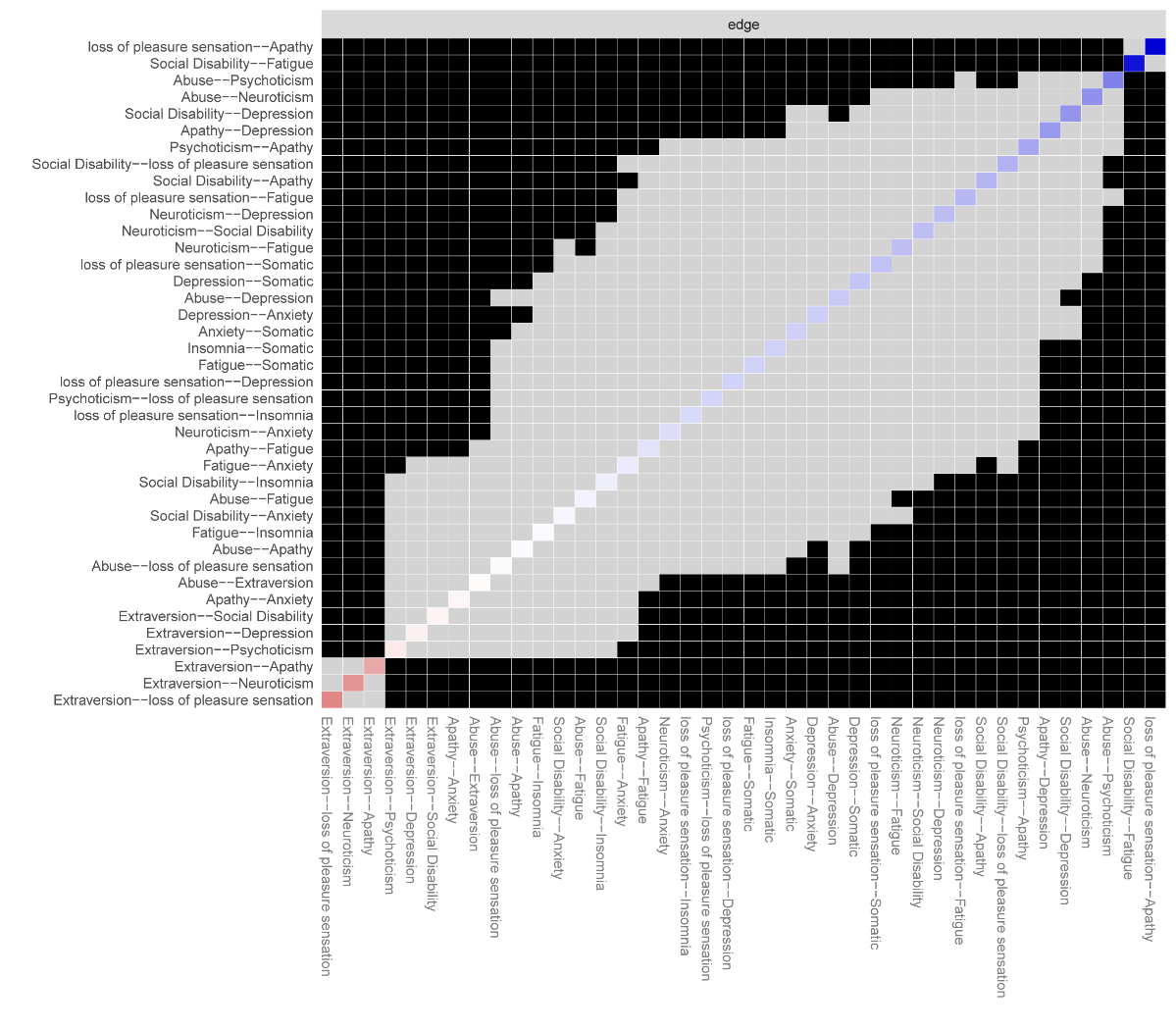


Figure S6. Edge weight differences at baseline

*Note: Edges that differ significantly from each other are depicted by black boxes.*


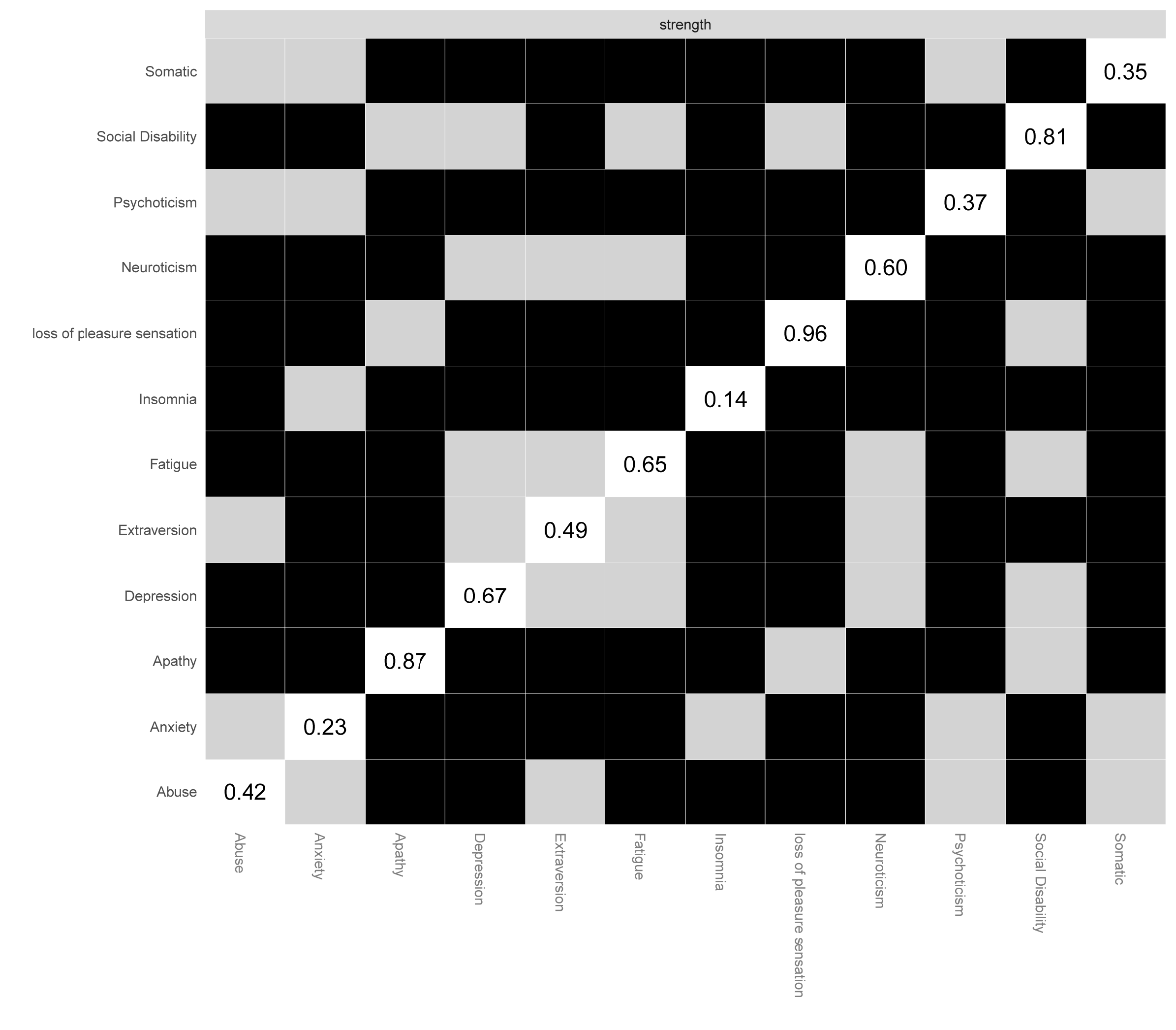


Figure S7. Strength differences at baseline

*Note: Nodes that differ from each other in strength, i.e. the combination of all edge weights connecting to that node. Black boxes represent significant differences, and strength values are in the diagonal.*

**Epskamp, S. & Fried, E. I.** 2018. A tutorial on regularized partial correlation networks. *Psychol Methods* 23(4), 617-634. <https://doi.org/10.1037/met0000167>
